# Vesicular Stomatitis Virus-Based Vaccines Provide Cross-Protection against Andes and Sin Nombre Viruses

**DOI:** 10.3390/v11070645

**Published:** 2019-07-13

**Authors:** Bryce M Warner, Derek R Stein, Rohit K Jangra, Megan M Slough, Patrycja Sroga, Angela Sloan, Kathy L Frost, Stephanie Booth, Kartik Chandran, David Safronetz

**Affiliations:** 1 Zoonotic Diseases and Special Pathogens, National Microbiology Laboratory, Public Health Agency of Canada, Winnipeg, MB R3E3R2, Canada; 2 Department of Medical Microbiology, University of Manitoba, Winnipeg, MB R3E0J9, Canada; 3 Department of Microbiology and Immunology, Albert Einstein College of Medicine, Bronx, NY 10461, USA

**Keywords:** Hantavirus, prophylactic immunization, vaccine, vaccination, hantavirus cardiopulmonary syndrome, Andes virus, Sin Nombre virus

## Abstract

Andes virus (ANDV) and Sin Nombre virus (SNV) are the main causative agents responsible for hantavirus cardiopulmonary syndrome (HCPS) in the Americas. HCPS is a severe respiratory disease with a high fatality rate for which there are no approved therapeutics or vaccines available. Some vaccine approaches for HCPS have been tested in preclinical models, but none have been tested in infectious models in regard to their ability to protect against multiple species of HCPS-causing viruses. Here, we utilize recombinant vesicular stomatitis virus-based (VSV) vaccines for Andes virus (ANDV) and Sin Nombre virus (SNV) and assess their ability to provide cross-protection in infectious challenge models. We show that, while both rVSVΔG/ANDVGPC and rVSVΔG/SNVGPC display attenuated growth as compared to wild type VSV, each vaccine is able to induce a cross-reactive antibody response. Both vaccines protected against both homologous and heterologous challenge with ANDV and SNV and prevented HCPS in a lethal ANDV challenge model. This study provides evidence that the development of a single vaccine against HCPS-causing hantaviruses could provide protection against multiple agents.

## 1. Introduction

Hantaviruses are a family of enveloped, tri-segmented, negative-sense RNA viruses that are part of the order Bunyavirales. They are zoonotic pathogens found mainly in murid and cricetid rodents, as well as moles, shrews, and bats, and have a worldwide distribution [1]. In humans, they are able to cause two distinct diseases. Hemorrhagic fever with renal syndrome (HFRS), caused by Old World hantaviruses mainly in Europe and Asia, is characterized by hemorrhagic manifestations and acute renal dysfunction and has a mortality rate ranging from <1% to 15% depending on the causative agent [1]. Hantavirus cardiopulmonary syndrome (HCPS) is caused by New World hantaviruses found in the Americas and is a severe cardiopulmonary disease characterized by respiratory failure, pulmonary edema, and cardiogenic shock, with fatality rates greater than 35% [1].

Sin Nombre virus (SNV) and Andes virus (ANDV) are both New World hantaviruses that are responsible for the majority of HCPS cases in North and South America, respectively [2,3]. SNV is carried by *Peromyscus maniculatus* (deer mice) and has been responsible for greater than 800 cases in North America since the virus was discovered in the early 1990s [2]. ANDV has a higher prevalence than SNV and is carried by the long-tailed pigmy rice rat, *Oligoryzomys longicaudatus*. The virus was identified in the mid-1990s following HCPS outbreaks in Argentina and Chile [3]. Humans become infected via inhalation of aerosolized virus found in the secreta and excreta left by infected rodents. There has also been documented person-to-person transmission of ANDV [4].

There are currently no FDA-approved treatments or vaccines for HFRS or HCPS. Ribavirin has had some success in treating HFRS and has been shown to protect Syrian hamsters in a lethal model of HCPS [5]. However, a clinical trial reporting on its efficacy during HCPS in humans was inconclusive [6]. Passive transfer of neutralizing convalescent serum was also investigated in a clinical setting and there is some evidence that this approach might have some benefit [7]. Much of the vaccine development for hantaviruses has focused on HFRS-causing viruses, including the rodent brain-derived inactivated vaccine Hantavax, which has been used extensively in Asia to protect against HFRS caused by Hantaan virus [8]. No vaccines against HCPS-causing viruses have advanced toward clinical trials. Several candidate vaccines have been tested in animal models for both immunogenicity and protective efficacy. A recombinant DNA vaccine expressing the SNV M segment was shown to be immunogenic and protective in both hamster and deer mouse models of infection [9,10]. However, the only lethal model of SNV infection in immunocompetent animals remains the non-human primate model and no vaccine studies have utilized this model to date. Multiple vaccines for ANDV have also been tested in animal models, including genetic vaccines and a recombinant vesicular stomatitis virus (VSV) expressing the ANDV glycoprotein precursor [11,12]. The latter successfully protected Syrian hamsters against lethal ANDV infection, a model that recapitulates the human course of disease [13].

Cross-protection against multiple hantavirus species via vaccination with a single vaccine has been an area of exploration for both HCPS-causing and HFRS-causing viruses. For example, DNA and vaccinia virus-vectored vaccines expressing the M segment of Hantaan virus (HTNV) were shown to protect not only against challenge with HTNV, but also with other HFRS-causing viruses, such as Seoul virus (SEOV), Puumala virus (PUUV), and Dobrava virus (DOBV) [14,15]. For HCPS, a DNA vaccine expressing ANDV M segment was shown to elicit antibodies against not only ANDV, but also SNV and Black Creek Canal virus, while vaccination against HTNV did not result in antibody production against any of the HCPS-causing viruses tested [16]. Vaccination of hamsters with a DNA vaccine encoding SNV M segment was also shown to be immunogenic, but was not able to protect hamsters from lethal ANDV challenge [10]. Similarly, passive transfer of immune sera from SNV-vaccinated rabbits was able to protect against homologous SNV challenge, but not against heterologous challenge with lethal ANDV, suggesting that cellular immunity may be important for vaccine-mediated cross-protection [17]. HFRS/HCPS dual vaccines have also been tested in pre-clinical models, with HFRS and HCPS DNA vaccines combined to produce a cross-clade-hantavirus vaccine. An HNTV/ANDV DNA vaccine expressing both HTNV and ANDV M segments was shown to elicit high antibody titers against both viruses [18]. Additionally, a mix of SNV, ANDV, HTNV, and PUUV DNA vaccines was administered to rabbits and was able to elicit a strong humoral response. However, the utility of this approach has not been assessed in infectious challenge models [10].

Here, we utilized recombinant VSV vaccines expressing SNV and ANDV glycoproteins (rVSVΔG/SNVGPC and rVSVΔG/ANDVGPC) and tested their ability to protect against both homologous and heterologous challenges in Syrian hamster models of ANDV and SNV infection/disease. Both the rVSVs were able to induce cross-reactive IgG responses in vaccinated hamsters, as assessed by ELISA and neutralization assays. Both vaccines were protective against lethal ANDV challenge and against non-lethal hamster-adapted SNV infection (HA-SNV) [19]. This provides evidence that a singular HCPS-preventing vaccine can protect against both SNV and ANDV.

## 2. Materials and Methods

### 2.1. Ethics Statement

The animal experiments described were carried out at the National Microbiology Laboratory (NML) of the Public Health Agency of Canada. All experiments were approved by the animal care committee at the Canadian Science Center for Human and Animal Health in accordance with guidelines provided by the Canadian Council on Animal Care. All animals were acclimated for at least one week prior to experimental manipulations. All infectious ANDV and SNV work was performed under biosafety level 4 (BSL-4) conditions at the NML. The animals were given food and water ad libitum and monitored daily throughout the course of the experiments.

### 2.2. Cells and Viruses

VeroE6 and Vero cells (African green monkey kidney, ATCC, CRL-1586 and CCL-81) were grown in Dulbecco’s modified Eagle’s medium (DMEM) (Hyclone, San Angelo, TX, USA) containing 2–10% fetal growth serum + penicillin–streptomycin (1000U) (Hyclone, San Angelo, TX, USA). Primary human umbilical vein endothelial cells (HUVEC) obtained from Lonza were maintained in endothelial growth media (EGM) supplemented with EGM-SingleQuots (Lonza, Basel, Switzerland). The VSV constructs used for kinetics and immunization have been described previously [11,20,21]. The ANDV strain Chile-9717869 was propagated on VeroE6 cells containing 2% fetal growth serum (FGS) and titered, as previously described [11]. Hamster-adapted Sin Nombre virus (HA-SNV) was passaged in Syrian golden hamsters, as previously described [19].

### 2.3. Growth Kinetics of VSV Constructs

An assay was performed on the following four viruses to assess growth kinetics: Wild type VSV, rVSVΔG/ANDVGPC, rVSVΔG/SNVGPC, and a VSV expressing Lassa virus glycoprotein (rVSVΔG/LASVGPC). Briefly, 12-well plates with VeroE6 cells at 80%–90% confluency were infected in triplicate with each virus at a multiplicity of infection (MOI) of 10^−4^. Following incubation at 37 °C for 1 h, media was replaced with 1 mL of DMEM + 2% FGS. Plates were incubated at 37 °C and supernatant was collected from each well at time points 0, 3, 6, 12, 24, 48, 72, and 96 hours. The supernatants collected at each time point were used for subsequent TCID_50_ analysis. Ten-fold serial dilutions of each supernatant were tested in triplicate in 96-well format in DMEM + 2% FGS. A mock-infected row was included per plate as a control. Plates were incubated at 37 °C and cytopathic effect (CPE) was monitored and recorded at 96 h post infection and TCID_50_’s were calculated using the Reed and Muench method.

### 2.4. Immunization of Hamsters and Challenge with ANDV or Hamster-Adapted SNV

For immunization, five to six week old female Syrian golden hamsters (Mesocricetus auratus) were anaesthetized with inhalational isoflurane and were given an intraperitoneal (IP) injection of 10^5^ plaque-forming units (PFU) of either rVSVΔG/ANDVGPC, rVSVΔG/SNVGPC, or rVSVΔG/LASVGPC. At 28 days post-immunization, hamsters were challenged IP with either 200 focus-forming units (FFU) of ANDV or the equivalent of 2 × 10^5^ genome copies of HA-SNV. Animals were monitored for clinical signs of disease, including hunched posture, labored breathing, and lethargy daily according to an approved scoring sheet. Animals requiring euthanasia due to clinical score or from a pre-determined experimental time point were exsanguinated via cardiac puncture after induction of deep anesthesia.

### 2.5. Detection of ANDV and SNV RNA

At days 4 and 7 post-infection, hamster tissues were collected and homogenized in 600 µL RLT lysis buffer (Qiagen, Hilden, Germany), clarified by centrifugation, diluted to 30 mg equivalents in RLT lysis buffer, and extracted using an RNeasy mini kit (Qiagen). RNA from serum was extracted using a Viral RNA mini kit (Qiagen). RT-qPCR detection of ANDV and SNV S segment RNA was performed on a StepOne Plus instrument (Applied Biosystems, Foster City, CA, USA) using a one-step protocol with a QuantiTect Probe RT-PCR (Qiagen) kit and SNV and ANDV-specific primers and probes (SNVforw—GCAGACGGGCAGCTGTG; SNVrev—AGATCAGCCAGTTCCCGCT; SNVProbe—5′FAM-TGCATTGGAGACCAAACTCGGAGAACTC-TAMRA; ANDVforw—AAGGCAGTGGAGGTGGAC; ANDVrev—CCCTGTTGGATCAACTGGTT; ANDVProbe—FAM-ACGGGCAGCTGTGTCTACATTGGA-TAMRA) according to manufacturer’s instructions. RT-PCR stages consisted of reverse transcription (50 °C for 30 min), Taq activation (95 °C for 15 min), and amplification (40 cycles at 94 °C for 15 s and 60 °C for 60 s). Data acquisition occurred at the end of the annealing/extension stage (60 °C for 60 s) of each amplification cycle. Samples were quantified against a standard curve of either ANDV or SNV S segment in vitro transcribed RNA ranging from 5 × 10^7^ to five S segment copies.

### 2.6. Anti-ANDV and Anti-SNV ELISA

For detection of ANDV and SNV glycoprotein-specific antibodies following immunization, 96-well half-area plates (Corning, Corning, NY, USA) were coated with 500 ng/well ANDV or SNV virus-like particles overnight at 4 °C. The following day, plates were washed three times with PBS-T and coated for 1 h with 5% skim milk + 0.1% tween 20. Following blocking, plates were washed three times with PBS-T and hamster serum diluted 1:100 in blocking buffer was added to plates in triplicate and incubated at 4 °C overnight. The next day, the plates were washed three times with PBS-T and secondary peroxidase-labelled anti-hamster IgG was added to the plates (1:1000) for 1 h at 37 °C. Following three washes with PBS-T, 75 µL/well of one-step ABTS substrate (Thermofisher, Waltham, MA, USA) was added to the plates for 30 min at room temperature. Plates were then read at 405 nm and analyzed using SoftMax Pro software (version 6.1).

### 2.7. Detection of ANDV and SNV Neutralizing Antibodies

Recombinant VSV bearing glycoproteins of either ANDV or SNV and expressing green fluorescent protein were incubated with dilutions of vaccinated hamster sera (20, 60, 180, 540, 1620 on Vero cells or 2-fold serial dilutions starting at 100 on HUVEC) for 1 h at room temperature (RT) and then used to infect cell monolayers in duplicate. Cells were scored for infection at 14 h post-infection via GFP expression by automated counting with a CellInsight CX5 fluorescence microscope and onboard HCS Studio software (Thermo Fisher). Percentage of relative infection was determined as compared to infection in the absence of serum. The Reed–Meunch method was used to calculate NT80, the titer of serum at which ≥80% reduction of relative infection is seen.

### 2.8. ANDV GPC-PCDH1 Binding Competition ELISA

The capacity of hamster sera to block binding of PCDH1 to ANDV GPC was evaluated as described previously [22]. Briefly, 100 ng per well of sEC1-2 (soluble EC1-2, the first two extracellular cadherin domains of PCDH1) was coated onto high-protein binding 96-well plates at 4 °C overnight and blocked with 5% nonfat dry milk in PBS. Pre-titrated amounts of FSL-biotin-labeled rVSV–ANDV GPC particles were then incubated with two-fold serial dilutions (starting at a 25-fold dilution) of hamster sera for 1 h at 37 °C before their application to the ELISA plates. Bound virus particles were detected by Streptavidin–HRP. Data from three independent experiments (average ±SD, *n* = 9) were expressed as % relative binding by setting no serum well-binding to 100%.

### 2.9. Histology

Hematoxylin and eosin staining was performed as described previously [23]. Briefly, formalin-fixed tissues were embedded in paraffin wax to make paraffin blocks. Five mm sections were cut and mounted on Superfrost microscope slides (Fisher, Ontario, Canada). Following an overnight incubation at 37 °C, sections were deparaffinized with three 5 min changes of xylene. Slides were then immersed three times in 100%, twice in 95%, and once in 70% ethanol for 3 min each. They were then washed with distilled water for 2 min and then stained for 2 min with hematoxylin (Richard Allen Scientific 7211, San Diego, CA, USA). A water rinse was performed for 2 min followed by differentiation in 1% acid alcohol treatment (8–12 dunks) and a second rinse in Scott’s tap water for 1 min followed by a rinse for 1 min. A 2 min counter stain was then performed in eosin Y (Surgipath, Richmond, IL, USA). Sections were dehydrated with two washes of 95% ethanol for 3 min each. A second set of washes was then performed three times in 100% ethanol for 3 min each and then cleared with three changes of xylene for 5 min each. Slides were mounted with Permount (Fisher) for viewing. Slides were scanned with a Zeiss Mirax Midi (Oberkochen, Germany).

### 2.10. Statistical Analysis

All results were analyzed and graphed using Prism 5 software (Graphpad, San Diego, CA, USA). Statistical significance between groups was determined using a Mann–Whitney test, one-way analysis of variance (ANOVA), two-way ANOVA, or Kaplan–Meier analysis with log-rank test, where applicable.

## 3. Results

### 3.1. Replication Kinetics of rVSVΔG/SNVGPC and rVSVΔG/ANDVGPC

**Figure 1:**
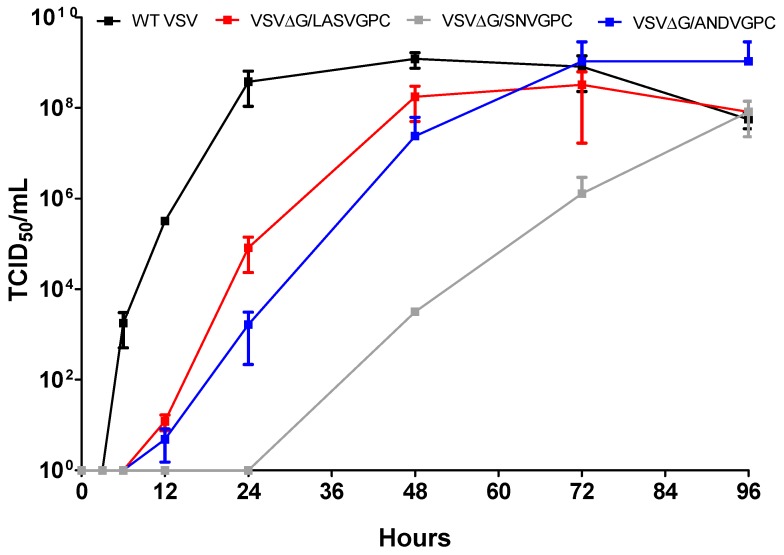
Growth kinetics of different recombinant vesicular stomatitis viruses (VSVs). Each virus was used to infect VeroE6 cells at MOI 10^−4^ for 96 h. The TCID50/mL of each virus is indicated for each time point. Data shown are mean + SD.

To determine if recombinant VSV viruses show altered rates of growth, TCID_50_ assays were performed using supernatant collected at different time points over a period of 96 h. As seen in Figure 1, VSV-WT showed the fastest growth rate when compared to rVSVΔG/ANDVGPC, rVSVΔG/LASVGPC, and rVSVΔG/SNVGPC. For VSV-WT, a CPE could be seen early with peak titers occurring at hour 48 and tapering off afterwards. Similar growth rates were seen with rVSVΔG/LASVGPC and rVSVΔG/ANDVGPC, both steadily increasing from hours 12 to 72. Although all three recombinant viruses exhibited slower growth rates when compared to wild type VSV, the most drastic difference was seen with rVSVΔG/SNVGPC. From hours 0–24 there was no detectable virus, followed by a slow growth increase from hours 24 to 72. At 72 h post-infection, high viral titers (up to 10^8^ TCID50) were seen for VSV-WT, rVSVΔG/LASVGPC, and rVSVΔG/ANDVGPC, while rVSVΔG/SNVGPC titers remained two logs below and did not reach 10^8^ TCID50 until 96 h post-infection. It was clear that the insertion of SNV glycoprotein in place of VSV glycoprotein significantly attenuated VSV replication kinetics.

### 3.2. Immunogenicity of VSVΔG/SNVGPC and VSVΔG/ANDVGPC

**Figure 2:**
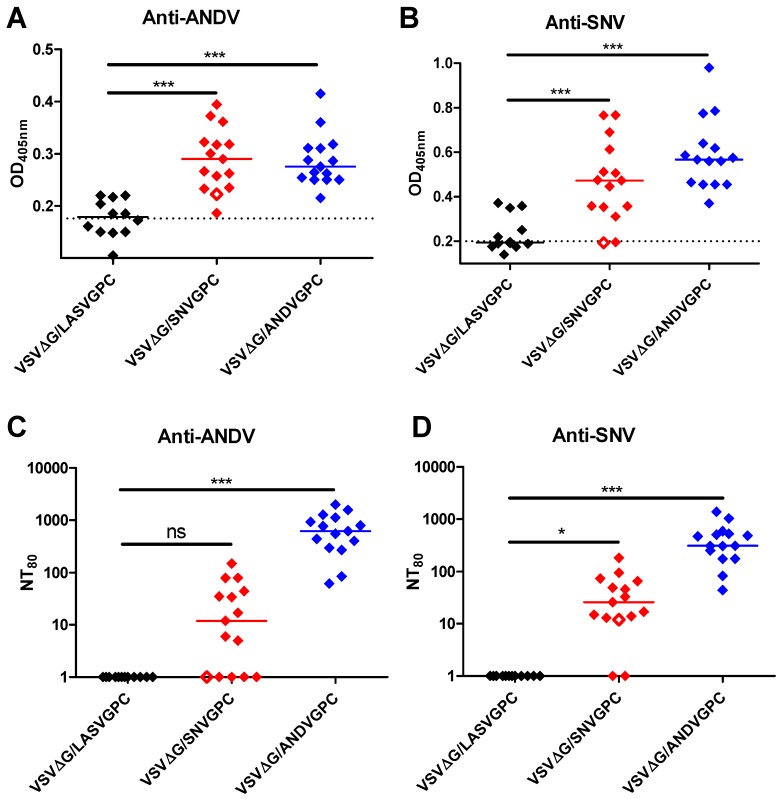
Humoral immune responses of vaccinated hamsters. Hamsters were vaccinated with either rVSVΔG/LASVGPC (*n* = 12), rVSVΔG/ANDVGPC (*n* = 15), or rVSVΔG/SNVGPC (*n* = 15) and IgG titers against either (**A**) ANDV or (**B**) SNV were assessed. NT80 against either (**C**) ANDV or (**D**) SNV was determined via microneutralization assay using recombinant VSV expressing either ANDV or SNV glycoprotein and GFP. Data medians are shown. Statistical significance was determined by one way ANOVA. *, *p* = <0.05; ***, *p* = <0.0001. Empty diamond represents a hamster that succumbed to ANDV infection. Black diamonds are rVSVΔG/LASVGPC vaccinated, red diamonds are or rVSVΔG/SNVGPC vaccinated, and blue diamonds are rVSVΔG/ANDVGPC vaccinated.

There is evidence that strong humoral responses against hantaviruses are important for protection against infection and disease [7,17]. To determine how well each vaccine is able to induce anti-ANDV and anti-SNV humoral responses, groups of hamsters were vaccinated with either rVSVΔG/ANDVGPC or rVSVΔG/SNVGPC and serum was collected from each animal 28 days post-vaccination. We assessed the IgG titers of each hamster against both ANDV and SNV via ELISA. Both vaccines elicited higher anti-ANDV and anti-SNV IgG titers than the control vaccination (Figure 2A,B). Interestingly, both vaccines elicited similar levels of antibody against both ANDV and SNV, with the rVSVΔG/ANDVGPC group even having slightly higher levels of anti-SNV antibody than the rVSVΔG/SNVGPC group, although this was not statistically significant. There is also evidence that the presence of neutralizing antibodies during hantavirus infection can protect individuals from developing severe disease [7]. Here, both vaccines were able to induce neutralizing antibodies as measured by NT80, defined here as the dilution of serum at which 80% relative infection is blocked, calculated by the Reed–Meunch method (Figure 2C,D). rVSVΔG/ANDVGPC appears to be able to induce a higher neutralizing titers than rVSVΔG/SNVGPC. This could be due to the attenuated replication of rVSVΔG/SNVGPC as compared to rVSVΔG/ANDVGPC. Vaccination with rVSVΔG/LASVGPC, which tends to replicate with greater efficiency than both hantavirus vaccines, also typically does not induce high neutralizing antibody titers following one vaccination, but the glycoprotein of LASV is also heavily glycosylated which may reduce the neutralization capacity, so it is not currently clear whether replication kinetics are directly correlated with immunogenicity [24]. Nevertheless, we have shown that both rVSV vaccines were able to induce humoral immune responses against ANDV and SNV, respectively.

**Figure 3:**
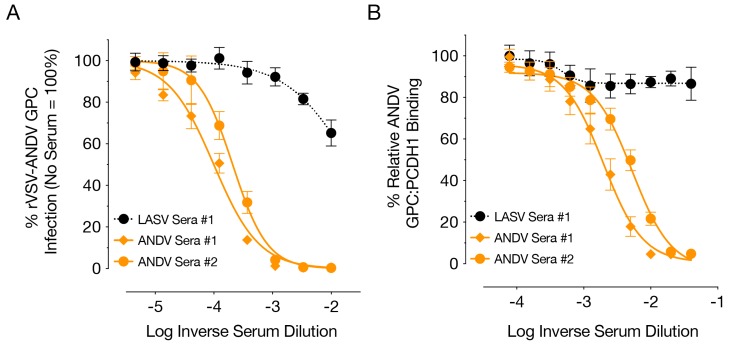
Blocking of ANDV GPC:PCDH1 interaction by hamster sera. (**A**) Neutralization activity of sera from vaccinated hamsters was assessed using primary human endothelial cells infected with rVSVΔG/ANDVGPC. Average ± SD (*n* = 6) from 3 independent experiments. (**B**) Ability of vaccinated hamster sera to block binding of ANDV GPC to soluble PCDH1 was assessed by competition ELISA. Mean ± SD (*n* = 9) from 3 independent experiments.

To gain further insight into the mechanism of neutralization, we tested the neutralization activity of a selected set of sera on primary human endothelial cells (HUVEC), which model infection of the major targets of hantavirus infection in vivo. Sera from rVSVΔG/ANDVGPC-immunized hamsters specifically neutralized ANDV GPC-mediated infection of HUVECs (Figure 3A) and also blocked binding of ANDV GPC to protocadherin-1 (PCDH1) (Figure 3B), the recently identified New World hantavirus receptor [22] suggesting that neutralizing antibodies block infection, at least partly, by preventing virus–receptor recognition.

### 3.3. rVSVΔG/ANDVGPC Immunization Provides Protection Against ANDV and HA-SNV

**Figure 4:**
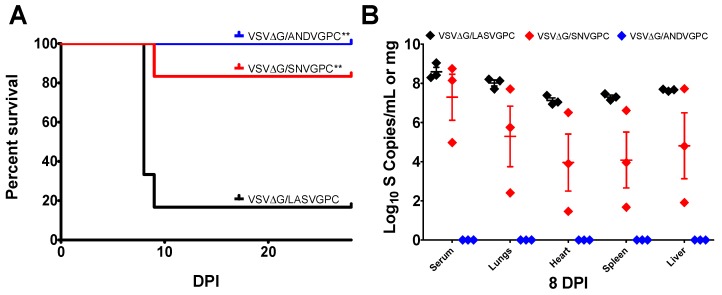
Protective efficacy of rVSVΔG/ANDVGPC and rVSVΔG/SNVGPC against ANDV challenge (**A**) Survival of hamsters vaccinated with either rVSVΔG/ANDVGPC (*n* = 6), rVSVΔG/SNVGPC (*n* = 6), or control vaccine rVSVΔG/LASVGPC (*n* = 6) following challenge with ANDV. (**B**) Presence, following ANDV challenge, of ANDV RNA in the serum and tissues in groups of vaccinated hamsters at 8 days post-infection (dpi) (*n* = 3). Data means + SEM are shown. Statistical significance was determined via log-rank test (**A**). **, *p* = <0.01.

**Figure 5:**
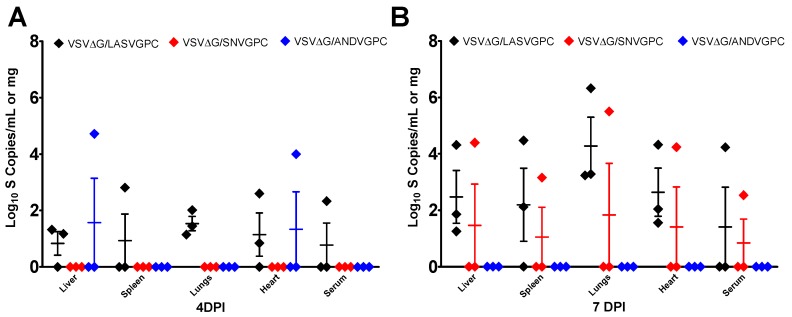
Protective efficacy of rVSVΔG/ANDVGPC and rVSVΔG/SNVGPC against HA-SNV challenge. Presence of SNV RNA in the serum and tissues of vaccinated hamsters at (**A**) 4 dpi and (**B**) 7 dpi (*n* = 3/group) following HA-SNV challenge. Data means + SEM are shown.

**Figure 6:**
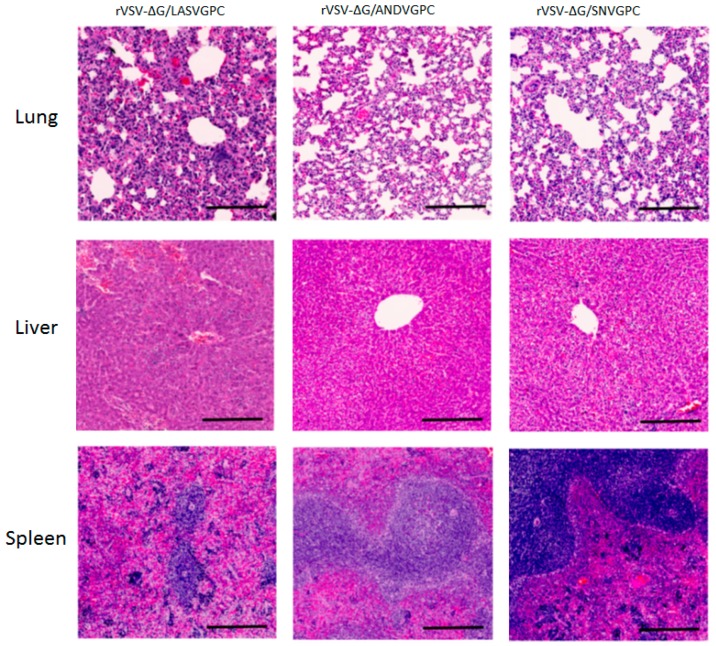
Histopathology of organs in vaccinated hamsters. Hematoxylin and eosin staining was performed on the lungs, livers, and spleens of hamsters on day 7 post-ANDV infection, following vaccination with either VSVΔG/LASVGPC, VSVΔG/ANDVGPC, or VSVΔG/SNVGPC. Scale bar = 200 µm.

To determine whether vaccination with rVSVΔG/ANDVGPC is able to confer protection against both ANDV and SNV, vaccinated hamsters were challenged with either ANDV or HA-SNV. As shown previously [11], rVSVΔG/ANDVGPC vaccination provides complete protection from lethal ANDV challenge (Figure 4A). No rVSVΔG/ANDVGPC vaccinated animals had any detectable ANDV RNA levels 8 days post-infection (dpi) in the serum or tissues (Figure 4B). rVSVΔG/ANDVGPC vaccination also reduced the amount of HA-SNV, with infected animals showing reduced levels of SNV RNA in the serum and tissues at 3 and 7 (dpi) as compared to control animals (Figure 5A,B). Only one hamster showed positive SNV RNA levels following HA-SNV challenge, at 3 dpi, and all rVSVΔG/ANDVGPC vaccinated animals showed no detectable SNV RNA at 7 dpi (Figure 5A,B). rVSVΔG/ANDVGPC vaccinated animals also had significantly reduced pathology in the tissues while rVSV-ΔG/LASVGPC vaccinated animals showed moderate to severe pulmonary edema, hemorrhaging and infiltration of mononuclear cells in the lungs, hepatocellular necrosis and infiltration of lymphocytes into the liver, as well as some infiltration of mononuclear cells into the red pulp of the spleen (Figure 6). Our data show that vaccination with rVSVΔG/ANDVGPC provides robust protection against both ANDV and SNV.

### 3.4. rVSVΔG/SNVGPC Immunization Provides Protection Against HA-SNV and ANDV

To assess whether rVSVΔG/SNVGPC is also able to provide protection against both ANDV and SNV infection, we vaccinated groups of hamsters with rVSVΔG/SNVGPC followed by challenge with either HA-SNV or lethal ANDV challenge. Similar to rVSVΔG/ANDVGPC, rVSVΔG/SNVGPC was able to protect hamsters against lethal ANDV challenge, with 83% surviving infection compared to 16% of controls (Figure 4A). The vaccine was also able to reduce pathology in the tissues, with little to no histological changes seen in the tissues sampled, similar to animals vaccinated with rVSVΔG/ANDVGPC (Figure 6). rVSVΔG/SNVGPC, however, did not reduce the amount of viral RNA in the tissues as seen with rVSVΔG/ANDVGPC vaccination. Animals receiving rVSVΔG/SNVGPC had significantly higher RNA levels in the serum and all tissues compared to rVSVΔG/ANDVGPC vaccinated animals and RNA levels were not significantly different than controls as assessed by two-way ANOVA (Figure 4B). For homologous challenge following rVSVΔG/SNVGPC vaccination, with the exception of a single animal at 7 dpi, all hamsters were negative for SNV RNA in the serum and tissues (Figure 5A,B), showing that the vaccine provides strong homologous immunity. While recombinant VSVs expressing SNV glycoproteins have been used for in vitro characterization of hantavirus biology [20], its efficacy as a vaccine against New World hantaviruses has not been explored. Here we show that a recombinant rVSVΔG/SNVGPC can provide protection against SNV infection as well as lethal ANDV infection.

## 4. Discussion

Currently there are no vaccines approved for protection against HCPS. While there has been a significant amount of focus on development of vaccines against HFRS, including the use in humans of an inactivated Hantaan virus vaccine, Hantavax, there has been little progress in the way of vaccines for HCPS [25,26]. DNA vaccines expressing the glycoproteins of ANDV and SNV have been developed and tested in preclinical models for immunogenicity [17,18]. Additionally, candidate vaccines for protection against multiple hantaviruses have been developed and tested, but their utility remains to be investigated in infectious models. The ability of vaccine candidates for New World hantaviruses to protect against multiple species has not been investigated. Here, we showed that recombinant VSV vaccines expressing ANDV or SNV glycoproteins could provide protection not only against homologous viral challenge, but also against heterologous challenge. The proportions of HCPS cases that these two viruses are responsible for in North and South America make them ideal agents against which to test the utility of cross-protective vaccines.

The substitution of the VSV glycoprotein for that of other viruses can often alter the replication ability of the newly formed viral particles. We wanted to determine whether the insertion of ANDV or SNV glycoproteins into VSV significantly affected the growth of the viruses in vitro. Both viruses showed delayed growth compared to wild type VSV, with VSVΔG/SNVGPC showing significantly attenuated growth kinetics (Figure 1). The molecular mechanisms behind this altered growth are unclear. Further investigation into the biology of SNV glycoprotein expression and packaging with VSV could shed some light on this, especially if there remain concerns about immunogenicity of this particular vaccine candidate.

While there remains a limited number of options to assess the immune response in hamsters [27], we assessed the humoral immunity generated by each vaccine. As seen in Figure 2, each vaccine was able to induce an IgG response, even though a number a hamsters did not generate neutralizing antibodies. Both vaccines were able to generate some neutralizing responses against both ANDV and SNV. Interestingly, the neutralizing antibody titers of each vaccinated group against both ANDV and SNV were similar (Figure 2), despite only about 77% identity in the amino acid sequences of their respective glycoproteins [28]. Part of the criteria for distinguishing hantavirus species is at least a four-fold difference in cross neutralization tests [28]. Therefore the similar homologous and heterologous titers seen could be due to the use of VSV constructs for both vaccination and neutralization assays, or the recognition of similar immune-dominant epitopes on the glycoproteins of ANDV and SNV in hamsters during antibody production. rVSVΔG/ANDVGPC was more immunogenic in terms of neutralizing antibody titers, which may be due to its reduced attenuation compared to the rVSVΔG/SNVGPC vaccine. Virus neutralization is mediated, at least in part, by blockage of binding of ANDV GPC to its receptor, PCDH1 (Figure 3). Other VSV vaccines expressing heterologous glycoproteins, such as that of Lassa virus do not readily induce neutralizing antibodies, which may be due in part to glycosylation of viral glycoproteins or the cellular tropism of the recombinant viruses [29]. While antibodies that are able to bind both ANDV and SNV were of particular interest here, the lack of suitable means to assess cell-mediated immunity induced by both vaccines makes it difficult to determine its possible role in protection. There is evidence that antibodies against one species of hantavirus alone are not sufficient to protect against other species in the absence of cell-mediated immunity [10]. Therefore, the ability of these vaccines to induce strong cell-mediated immunity is something that could be assessed in future studies.

Ultimately, the efficacy of both rVSV vaccines in protection against infectious challenge was investigated. Both vaccines were able to provide protection against both viruses, suggesting that the utility of a single vaccine expressing just one New World hantavirus glycoprotein could provide protection against multiple species. rVSVΔG/ANDVGPC completely protected hamsters against lethal ANDV challenge, as expected and reported previously [11], and was also able to largely protect against HA-SNV challenge with SNV RNA being detected in only one of the challenged hamsters (Figure 4 and Figure 5). While one rVSVΔG/SNVGPC-vaccinated animal succumbed to ANDV challenge, this vaccine was also able to significantly protect hamsters against lethal ANDV infection as compared to control-vaccinated animals, despite the presence of detectable ANDV in the tissues and serum. Similar to rVSVΔG/ANDVGPC-vaccinated animals, SNV RNA was detected in only one of the HA-SNV challenged hamsters on day 7, suggesting that this vaccine is also effective against SNV challenge.

The main limitation of the experiments conducted here is the lack of an appropriate immunocompetent lethal small animal model of SNV challenge. The only current lethal small animal model for SNV is an immunocompromised Syrian hamster model [30]. The use of this model for a vaccination study of this kind is inappropriate due to the possible effects of cell-mediated immunity needed to mediate protection against HCPS. Therefore, we decided to use HA-SNV to assess the efficacy of each vaccine against SNV. While this model suggests that each vaccine can provide protection, testing of these vaccines in a lethal model of SNV, such as a non-human primate model, is warranted.

To summarize, we determined the ability of recombinant VSV expressing ANDV or SNV glycoproteins to protect against ANDV and SNV challenges in Syrian hamster models of infection. Each vaccine is able to induce cross-reactive IgG responses and protect against both homologous and heterologous challenge. The ability of these vaccines to provide protection against multiple New World hantavirus species provides evidence that there is significant cross-reactivity between species, such that the design and development of one vaccine candidate could provide protection against multiple New World hantaviruses. This is an avenue that has been suggested, but has not been explored in infectious models previously. We show that the implementation of a single vaccine against HCPS-causing hantaviruses could provide beneficial protection against the two most prevalent species that can cause HCPS. With the previous development of a lethal NHP model of HCPS, further pre-clinical development of these vaccines is warranted.
